# PCR–RFLP assays for the identification of *Anopheles* (Diptera: Culicidae) species circulating in Honduras

**DOI:** 10.1186/s12936-023-04494-6

**Published:** 2023-02-18

**Authors:** Denis Escobar, Fernando Pérez, Bryan Ortiz, Gustavo Fontecha

**Affiliations:** Microbiology Research Institute, Universidad Nacional Autónoma de Honduras, Tegucigalpa, Honduras

**Keywords:** *Anopheles* spp., PCR–RFLP, Malaria, Honduras

## Abstract

**Background:**

Vector populations are a key target for malaria control and elimination. In Honduras, there are at least 12 reported anopheline species, however, the definitive number of species remains uncertain. Due to the inherent limitations of morphological identification of *Anopheles* species, molecular approaches have been developed to provide accurate identification and robust surveillance of local malaria vectors. The aim of this study was to design and assess three PCR–RFLP assays to identify anopheline species known to presently occur in Honduras.

**Methods:**

Mosquitoes captured between 2018 and 2022 in seven malaria-endemic and non-endemic departments in Honduras were analysed. The ITS2 ribosomal region and three restriction enzyme-based assays were evaluated in silico and experimentally.

**Results:**

A total of 132 sequences from 12 anopheline species were analysed. The ITS2 marker showed length polymorphisms that generated products between 388 and 592 bp and no relevant intraspecies polymorphisms were found. Furthermore, the three PCR–RFLP assays were able to differentiate 11 species with sufficient precision and resolution.

**Conclusion:**

The ITS2 region was shown to be a useful molecular marker for identifying local *Anopheles* species. In addition, the PCR–RFLP assays evaluated here proved to be capable of discriminating most of the anopheline species present in Honduras. These methods provide alternatives to improve entomological surveillance of *Anopheles* in Honduras and other Mesoamerican countries.

## Background

*Anopheles* mosquitoes are the only vectors of human malaria and are the main target of national programmes to achieve its control and elimination [1, 2]. *Anopheles* is a highly biodiverse genus with more than 500 recognized species and others yet to be characterized, particularly cryptic species grouped in complexes [3, 4]. Approximately 70 *Anopheles* species have been incriminated as vectors of *Plasmodium* spp. transmitting the parasite to humans [3]. *Anopheles* species have adapted to the ecological conditions of almost all regions of the world and their distribution is highly heterogeneous. Likewise, malaria parasites have adapted to survive immunological challenges within the vector species with which they interact in each region [5, 6].

The definitive distribution and number of *Anopheles* species in Honduras have not yet been determined, although several sources report the occurrence of 12 to 14 anopheline species in the country, however, none of them fully agree. According to reports from the Ministry of Health of Honduras and based on entomological surveillance activities in the last 10 years [7–9], there are 14 native anopheline species: four of the subgenus *Nyssorhynchus* (*Anopheles albimanus*, *Anopheles darlingi*, *Anopheles argyritarsis*, *Anopheles albitarsis*), nine of the subgenus *Anopheles* (*Anopheles vestitipennis*, *Anopheles pseudopunctipennis*, *Anopheles punctimacula*, *Anopheles crucians*, *Anopheles apicimacula*, *Anopheles neomaculipalpus*, *Anopheles gabaldoni*, *Anopheles grabhamii*, *Anopheles eiseni*), and one of the subgenus *Kerteszia (Anopheles neivai*) is the only representative of the subgenus (information taken from the reports made by the technicians in charge of entomological surveillance of the Honduran Ministry of Health). All these species have been described as malaria vectors in the Neotropics, but Honduras's dominant vector species is *An. albimanus* [3].

On the other hand, the database of The Walter Reed Biosystematics Unit (WRBU) [10] and the taxonomic key of female anophelines from Central America and Mexico [8] report 13 species present in Honduras, but six of them are not consistent with those described by the national authorities [11]. *Anopheles albitarsis*, *An. gabaldoni*, *An. grabhamii* and *An. neivai* are not included in the WRBU or taxonomic key, while *Anopheles bradleyi*, *Anopheles hectoris* and *Anopheles strodei* are included in the WRBU/taxonomic key but have not been reported by local entomologists. Added to this, three species are not included in the ‘Taxonomy’ tool of the National Centre for Biotechnology Information (NCBI) (*An. gabaldoni*, *An. grabhamii*, and *An. hectoris*). The NCBI taxonomy includes organism names and classifications for each sequence in the nucleotide and protein sequence databases of the International Nucleotide Sequence Database Collaboration (INSDC) and provides a framework for grouping elements within other domains for links to external web resources specific to relevant taxa and publications [12]. Also, three species have no available sequences for the internal transcribed spacer (ITS2) in the NCBI database (*An. bradleyi*, *An. eiseni*, and *An. strodei*).

Finally, the parasitology research group of the National University captures and identifies anophelines since 2018 in seven of the 18 departments of the country (endemic and non-endemic for malaria) and at this stage has captured and identified 10 species of *Anopheles* (Table 1) [7–9]. These discrepancies could reflect the limitations of the taxonomy of some native species, as well as the difficulty or impossibility of distinguishing anophelines based exclusively on morphological characteristics [13–15]. Morphological identification has demonstrated some disadvantages such as being time-consuming and requiring trained specialists; added to this, the defining anatomical structures are not always available or in good condition [16].

**Table 1:** Geographical coordinates of the collection sites and *Anopheles* species captured in each department

Department	Coordinates (Latitude, Longitude)	Altitude (masl)	*Anopheles* species collected and identified based on morphology
Atlántida	15.748587, − 86.900546 15.758790, − 86.867092	7	*An. albimanus* *An. darlingi* *An. vestitipennis* *An. punctimacula* *An. neivai*
Bay Islands	16.327423, − 86.536481	60	*An. albimanus*
Comayagua	14.439279, − 87.689953 14.651083, − 87.608472	430—640	*An. albimanus* *An. pseudopunctipennis* *An. argyritarsis*
Colón	15.938416, − 85.058888 15.773889, − 85.134556 15.629846, − 86.287587 15.655448, − 86.04725	5—80	*An. albimanus* *An. darlingi* *An. pseudopunctipennis*
Cortés	15.289617, − 88.029439 15.289617, − 87.977116 14.85536, − 87.929549	47—740	*An. albimanus*
El Paraíso	14.103168, − 86.917882	600	*An. albimanus*
Gracias a Dios	15.018379, − 83.641264 15.309131, − 83.565868 14.94412734, − 83.84507528 15.25098087, − 83.82883771 15.21331402, − 83.77352977	7—35	*An. albimanus* *An. vestitipennis* *An. crucians* *An. neivai* *An. argyritarsis* *An. apicimacula* *An. neomaculipalpus* *An. punctimacula*

These limitations have led to the development and incorporation of molecular tools that facilitate and speed up the identification of vectors in areas of high diversity. Multiplex PCR, allele-specific PCR and sequencing have been successfully used to diagnose *Anopheles* species in several countries [17–31]. Other assays are based on the digestion with restriction enzymes of DNA sequences such as ITS2, the cytochrome c oxidase subunits I and II, and the D3 region of the nuclear 28S rDNA gene [21, 32–39]. In the Americas, for example, Vezenegho et al. [32] were able to discriminate between 15 anopheline species from French Guiana using a combination of two restriction enzymes; in Colombia, Zapata et al. [35] managed to diagnose seven species with a single restriction enzyme; and Cienfuegos et al. [40] found congruent morphological and molecular identification results of four *Anopheles* species collected in northern and western Colombia using several restriction enzymes.

The PCR–RFLP is an easy and inexpensive approach compared to Sanger sequencing, which allows a large number of individuals to be processed, even if the complete insect is not available. In addition, it allows the identification of the immature stages of the mosquito, usually lacking discriminative morphological characteristics. In this study, three PCR–RFLP assays were developed and tested to easily identify 11 of the 12 most common anopheline species reported to occur in Honduras.

## Methods

### Mosquito collection

**Fig. 1:**
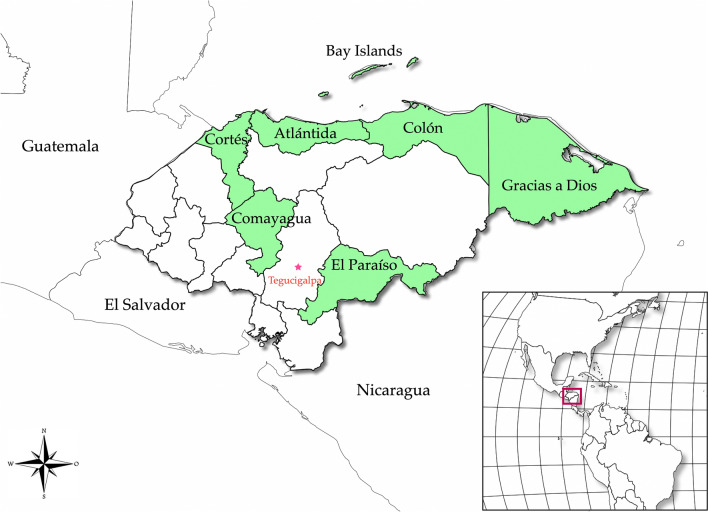
Map of Honduras showing the seven departments where mosquitoes were collected

Anopheline mosquitoes were captured between 2018 and 2022 in seven departments of Honduras: Gracias a Dios, Colón, Bay Islands, Comayagua, El Paraíso, Atlántida, and Cortés (Fig. 1). The geographical coordinates and the species collected in each department are shown in Table 1. The mosquitoes were collected in the peridomicile and extradomicile during the night. Two collection methods were used: the Center for Disease Control trap (CDC-LT) [41] provided with light as an attractant, and aspiration of resting mosquitoes, sometimes complemented by human landing catch. Mosquitoes were separated and stored dry in 1.5 mL tubes and subsequently identified under a stereoscope using morphological keys for female anophelines from Central America and Mexico [11].

Specimens of *An. gabaldoni* included in this study originate from Mexico (municipality of Othón P. Blanco, Quintana Roo), because none could be captured within Honduran territory over the study time frame. Additionally, it was not possible to have any specimen of *An. albitarsis*, thus molecular analyses were based on two sequences downloaded from the NCBI database (Acc. Numbers AF462385.1, AF462387.1) and uploaded by researchers from Brazil.

### DNA extraction

Most of the mosquitoes’ DNA analysed in this study came from the DNA bank of the Genetic Research Centre of the National University of Honduras [7, 9]. For those *Anopheles* species for which DNA was not available, genomic DNA was extracted from freshly collected specimens. Both the DNA of the historical specimens and that of the mosquitoes captured for this study were individually extracted following the protocol of the DNeasy Blood and Tissue Kit® (QIAGEN, Hilden, Germany). The heads and thorax of each mosquito were dissected. Single maceration was carried out using a pestle and a 1.5 mL conical tube following manufacturer’s instructions. Overnight lysis at 56 °C was carried out. DNA was eluted in 150 µL of elution buffer and stored at − 20 °C until further use.

### PCR

In order to assess whether it was possible to molecularly differentiate between the *Anopheles* species described in Honduras, the nuclear ribosomal internal transcribed spacer 2 (ITS2) was amplified [42]. PCR reactions were performed using the universal primers: 5.8S (5′-ATC ACT CGG CTC GTG GAT CG-3′) and 28S (5′-ATG CTT AAA TTT AGG GGG TAG TC-3′). The enzymatic reactions contained the following reagents: 25 μL of Taq Master Mix 2X (Promega Corp., Madison, WI, USA), 2 μL of each primer (10 μM), 2 μL of DNA (40 ng/μL), and 19 μL of nuclease-free water for a total reaction volume of 50 μL. PCR amplifications were performed under the following conditions: 94 °C for 5 min, 34 cycles of 94 °C for 30 s, 57 °C for 30 s, 72 °C for 30 s, and a final extension cycle of 72 °C for 10 min. Successful amplifications were confirmed on a 1.5% agarose gel with ethidium bromide. The size of the amplification products was assessed by comparison with a 100 bp ladder. Amplicons were sequenced in both directions using the same PCR primers. Sanger sequencing and purification services were provided by Psomagen (https://www.psomagen.com).

### *In-silico* analyses

The sequences were trimmed and analysed with the Geneious® 9.1.7 software (Biomatters Ltd, Auckland, New Zealand). Both strands were aligned, and a consensus sequence was obtained for each specimen. Consensus sequences were edited so that they were delimited by the same primers with which they were amplified. The size of the amplicons by species was determined and subjected to three *in-silico* restriction analyses with the enzymes AluI (AGˆCT), DdeI (CˆTNAG), and a combination of AluI with MseI (TˆTAA), as described elsewhere [32, 33, 35] using the Geneious® 9.1.7 software. Restriction patterns were established for each *Anopheles* species included in this study, and the intraspecific variability was evaluated.

### PCR–RFLP

To validate the prediction of the *in-silico* restriction profiles, experimental restriction assays were performed by digesting the ITS2 amplicons of each specimen with the above-mentioned enzymes: AluI (Promega, Corp.), DdeI (Promega, Corp.), and AluI/MseI (Thermo Scientific). The enzymatic reactions were carried out in a final volume of 20 μL, containing 2 μL of buffer (Tango buffer, Thermo Scientific for AluI and MseI, and buffer D for DdeI), 0.5 μL of each enzyme at a concentration of 10 units/μL, 10 μL of the PCR product, and 7 µL of nuclease-free water. The microtubes were incubated at 37 °C for 4 h and the digestion products were separated on a 2.5% agarose gel running at 70 V for 90 min. A molecular weight ladder of 100 bp was used. Restriction patterns were recorded on an image analyzer under ultraviolet light.

## Results and discussion

As shown in Table 2, the size of the amplification products of the ITS2 region ranged between 388 and 592 bp. These size polymorphisms could be sufficient to distinguish some anopheline species, such as *An. crucians*, *An. punctimacula*, and *An. neomaculipalpus* with products of 388, 445, and 504 bp, respectively. However, the size differences between *An. albimanus* and *An. pseudopunctipennis* (561–565 bp), and between *An. argyritarsis*, *An. albitarsis*, *An. apicimacula* and *An. gabaldoni* (526–536 bp), as well as between *An. darlingi*, *An. vestitipennis* and *An. neivai* (583–592 bp), are not enough for a clear diagnosis of each of the species [35].

**Table 2:** The number of specimens per species analysed by Sanger sequencing, amplicon sizes, and restriction patterns

Species	Number of specimens	ITS2 amplicon size (bp)	Predicted fragments sizes (bp)		
			AluI/MseI	AluI	DdeI
*An. (Nyss.) albimanus*	76	561	268, 162, 91, 33, 7	366, 162, 33	370, 112, 85
*An. (Nyss.) darlingi*	8	592	197, 172, 106, 77, 33, 7	303, 172, 84, 33	392, 112, 95
*An. (Nyss.) argyritarsis*	2	526	240, 157, 89, 33, 7	336, 157, 33	417, 112
*An. (Nyss.) albitarsis*	2*	536	242, 143, 85, 33, 26, 7	267, 150, 84, 33	431, 108
*An. (An.) vestitipennis*	14	583	247, 147, 80, 69, 33, 7	253, 216, 80, 33	317, 270
*An. (An.) pseudopunctipennis*	7	565	525, 33, 7	528, 33	301, 150, 117
*An. (An.) punctimacula*	2	445	216, 77, 71, 47, 33, 7	336, 76, 33	445
*An. (An.) crucians*	13	388	213, 80, 55, 33, 7	213, 80, 62, 33	228, 164
*An. (An.) apicimacula*	1	533	228, 120, 80, 42, 34, 7	372, 80, 47, 34	408, 118, 13
*An. (An.) neomaculipalpus*	3	504	248, 120, 80, 33, 16, 7	255, 136, 80, 33	504
*An. (An.) gabaldoni*	2	532	272, 140, 80, 33, 7	419, 80, 33	532
*An. (Kert.) neivai*	2	583	247, 147, 80, 69, 33, 7	254, 216, 80, 33	316, 270

*Anopheles albitarsis* specimens were not available. ITS2 sequences were downloaded from NCBI

A heterogeneous number of individuals of each species were sequenced (from 1 to 76) as shown in Table 2. The sequences were aligned to determine the number of intraspecific polymorphic sites. Polymorphic sites were identified in three species (three for *An. vestitipennis* and *An. pseudopunctipennis,* and six for *An. albitarsis*). The remaining eight species (excluding *An. apicimacula*) had 100% identical intraspecific sequences (Table 2). None of the polymorphisms found in the three species matched any target of the three restriction enzymes used in this study. This result suggests the reproducibility of the methods regardless of the intraspecific diversity of the mosquitoes. However, considering the low number of specimens for some of the species, it is necessary to be cautious since the real diversity of the ITS2 marker could be under-represented in the present study. Although the ITS2 region generally shows low intraspecific variation, making it suitable for the identification of closely related *Anopheles* species [43], future studies, including more specimens collected from a larger geographic area, may confirm the robustness of the techniques proposed here.

**Fig. 2:**
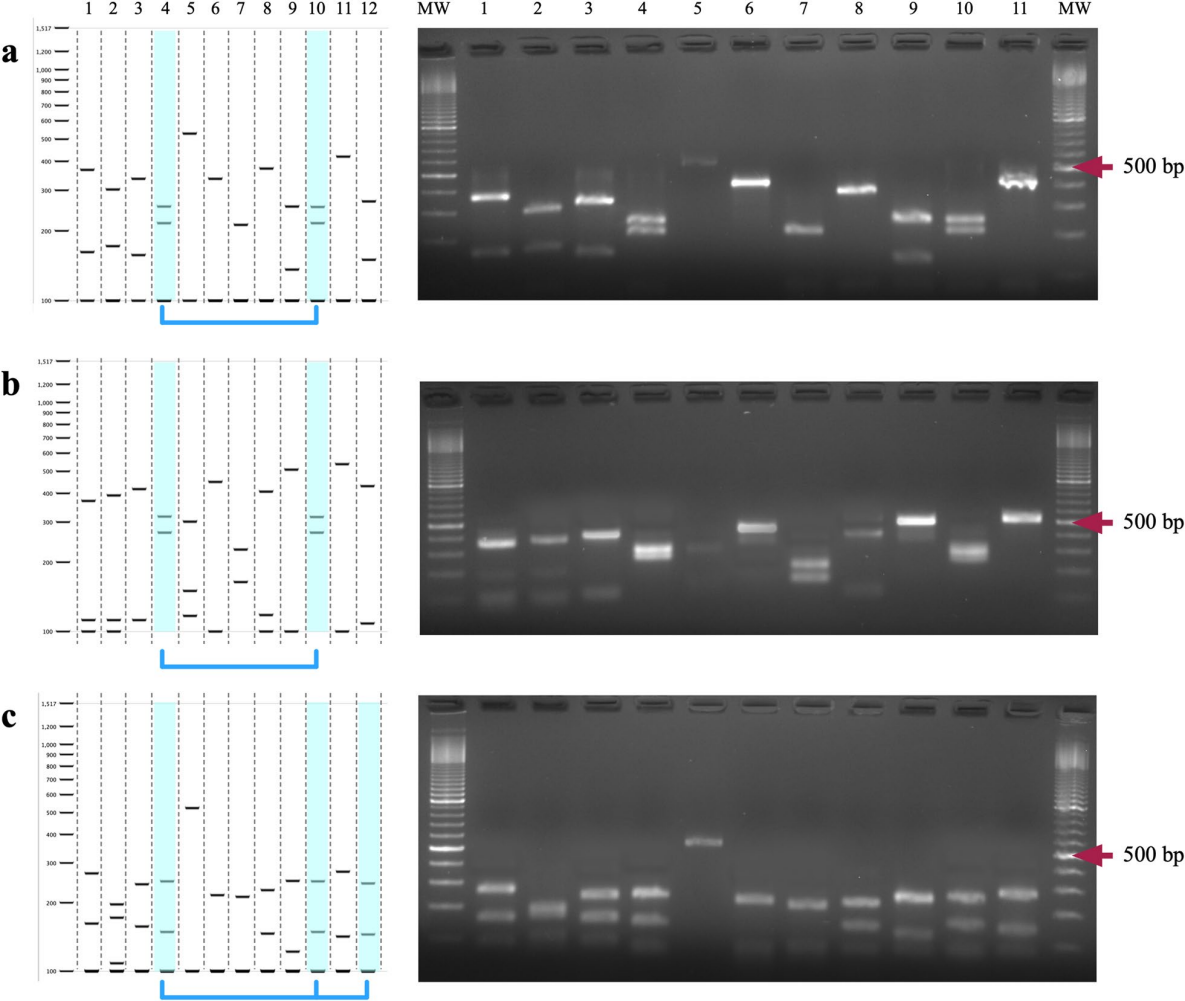
*In-silico* and experimental analysis of PCR–RFLP using the enzymes **a** AluI, **b** DdeI and **c** AluI and MseI. Lanes 1 to 12 correspond to *An. albimanus*, *An. darlingi*, *An. argyritarsis*, *An. vestitipennis*, *An. pseudopunctipennis*, *An. punctimacula*, *An. crucians*, *An. apicimacula*, *An. neomaculipalpus*, *An. neivai*, *An. gabaldoni*, and *An. albitarsis*. The restriction profile of *An. albitarsis* is not shown on agarose gels. A molecular weight marker of 100 bp was used

All three assays tested in the present study showed consistent results, with unique species-specific digestion patterns allowing for the differentiation of 10 of the anopheline species (Fig. 2). Despite this, some patterns can be difficult to distinguish in practice. The assay with AluI might be insufficient to distinguish between *An. albimanus* (366, 162 bp) and *An. argyritarsis* (336, 157 bp), as well as between *An. neomaculipalpus* (255, 136 bp) and *An. albitarsis* (267, 150 bp) even in gels with a higher concentration of agarose. Determining the size of the amplification product together with analysing the AluI restriction pattern would resolve the issue in both above-mentioned cases (Table 2). The use of two of the three PCR–RFLP assays could also help to resolve those cases in which there were doubts. The same situation occurs in the profiles generated by DdeI for *An. argyritarsis* (417, 112 bp), *An. apicimacula* (408, 118 bp), and *An. albitarsis* (431, 118 bp), but in this case, the size of the amplicon does not resolve the conflict. Two species still exhibited a potentially confounding pattern in the two-enzyme assay (AluI and MseI). *Anopheles argyritarsis* (240, 157 bp), *An. apicimacula* (228, 120 bp) and the amplification product size do not differ between species either. In addition to this, the pattern of *An. neivai*/*An. vestitipennis* (247, 147 bp) is too similar to the pattern of *An. albitarsis* (242, 143 bp).

Finally, none of the tested assays allowed to differentiate between *An. vestitipennis* and *An. neivai*. When comparing the sequences of the ITS2 region of both species, no diagnostic SNPs was found, showing 579 of 583 identical sites (99.1%). This was an unexpected finding, as both species belong to different subgenera (*An. (An.) vestitipennis vs An. (Kert.) neivai*) and are not considered cryptic species. In fact, both species are relatively easily differentiated according to the presence, distribution and colour of the scales on the thorax and abdomen, and the patterns of spots on the femora, tibiae and scutum, among other morphological structures [11].

Fourteen sequences of the COI gene of both species’ specimens collected previously in Honduras were further compared [7, 9]. These COI sequences showed that 611 of 627 nucleotides were identical (98.7%) between the two species. Similar or identical COI sequences have been shown in other insect taxa [16], confirming the difficulties inherent in the taxonomy of arthropods, with a high fraction of species or complexes yet to be defined. This challenge is aggravated by the scarce availability of records of specimens for some species. This limitation may be due to the fact that *An. neivai* and *An. vestitipennis* are distributed in poorly studied regions of the Neotropics. Consequently, the DNA sequence reference database is poorly developed and might not reflect the intraspecific genetic diversity of the species, which restricts barcoding gap analyses. Other molecular markers should be analysed to unravel the situation.

## Conclusions

Overall, the ITS2 region confirms to be an informative DNA fragment with low intraspecific variability. The three PCR–RFLP assays appear to be useful in discriminating most of the anopheline species distributed in Honduras and neighbouring Mesoamerican countries and, therefore, could be useful to medical entomologists in Southern Mexico and Central America as a tool to identify mosquitoes in the context of vector control activities. However, the AluI enzyme has the highest resolving power when the restriction profiles are analysed together with the size of the amplification product of ITS2. A combination of two or three trials might be advisable in cases where the restriction patterns of a single trial are not conclusive.

## Data Availability

Not applicable.

## Abbreviations

CDC: Centers for Disease Control and Prevention
ITS2: Nuclear ribosomal internal transcribed spacer 2
MASL: Metres above sea level
PCR–RFLP: Polymerase chain reaction-restriction fragment length polymorphism
SNPs: Single nucleotide polymorphisms
